# Comparative Immunogenicity of a Cytotoxic T Cell Epitope Delivered by Penetratin and TAT Cell Penetrating Peptides

**DOI:** 10.3390/molecules200814033

**Published:** 2015-08-03

**Authors:** Nicole Brooks, Sandra Esparon, Dodie Pouniotis, Geoffrey A. Pietersz

**Affiliations:** 1 School of Medical Sciences, RMIT University, Plenty Road, Bundoora 3083, Victoria, Australia; E-Mails: brooksy_4567@hotmail.com (N.B.); dodie.pouniotis@rmit.edu.au (D.P.); 2 Bio-Organic and Medicinal Chemistry Laboratory, Centre for Biomedical Research, Burnet Institute, 85 Commercial Rd, Melbourne 3004, Australia; E-Mail: sesparon@burnet.edu.au; 3 Department of Pathology, University of Melbourne, Parkville 3010, Victoria, Australia; 4 Department of Immunology, Monash University, Clayton 3800, Victoria, Australia

**Keywords:** TAT, penetratin, CPP, membrane translocating peptide, membrane penetrating peptide, vaccine, cytotoxic T cell epitope, immunogenicity, antigen presentation, antigen delivery, immunotherapy

## Abstract

Cell penetrating peptides (CPP), including the TAT peptide from the human immunodeficiency virus transactivator of transcription (HIV-TAT) protein and penetratin from *Drosophila Antennapedia* homeodomain protein, translocate various cargos including peptides and proteins across cellular barriers. This mode of delivery has been harnessed by our group and others to deliver antigenic proteins or peptides into the cytoplasm of antigen processing cells (APC) such as monocyte-derived dendritic cells (MoDC). Antigens or T cell epitopes delivered by CPP into APC *in vivo* generate antigen-specific cytotoxic T cell and helper T cell responses in mice. Furthermore, mice immunised with these peptides or proteins are protected from a tumour challenge. The functional properties of CPP are dependent on the various cargos being delivered and the target cell type. Despite several studies demonstrating superior immunogenicity of TAT and Antp-based immunogens, none has compared the immunogenicity of antigens delivered by TAT and Antp CPP. In the current study we demonstrate that a cytotoxic T cell epitope from the mucin 1 (MUC1) tumour associated antigen, when delivered by TAT or Antp, generates identical immune responses in mice resulting in specific MUC1 T cell responses as measured by *in vivo* CTL assays, IFNγ ELISpot assays and prophylactic tumour protection.

## 1. Introduction

Fundamental to an effective vaccine is the delivery of antigens to antigen presenting cells (APC) and the ensuing processing and presentation of epitopes to cytotoxic T cells or helper T cells in the context of a relevant MHC haplotype to activate T cells and induce an immune response [1,2]. Vaccination with peptides incorporating CTL epitopes has proven limited for a number of reasons including the inability of exogenous antigens to be presented efficiently to T cells [3,4]. There are now several strategies to promote the delivery of antigens to APC, such as targeting cell surface receptors with carbohydrates or antibodies [5,6,7,8,9,10]. We and others have utilised the unique translocating properties of cell penetrating peptides (CPP) to deliver antigenic proteins or T cell epitopes to APC utilizing covalent conjugates or synthetic tandem fusion peptides [11,12,13,14,15,16,17].

CPP offer a unique approach for the transport of peptides and proteins into the cytoplasm of APC. The TAT peptide (RKKRRQRRR) from the HIV transactivator of transcription protein and penetratin (Antp, RQIKIWFQNRRMKWKK) from *Drosophila Antennapedia* homeodomain are the two most widely investigated CPP [18]. Using dendritic cells pulsed with HIV TAT sequence with the tyrosinase related protein 2 (TAT-TRP-2), Wang and colleagues have shown that immunisation ensued complete protective immunity along with significant inhibition of lung metastases in a three day tumour model [19]. TAT, incorporating fusion proteins with CEA or MUC1, has also been used [20,21]. We have extensively used Antp in our studies of vaccine constructs for the delivery of intact proteins, such as ovalbumin (OVA) and mucin 1 (MUC1), as well as peptides comprised of single cytotoxic T cell or helper T cell epitopes or multiepitope peptides consisting of CD4 and CD8 epitopes [11,12,14,15,16,18]. Mice immunised with these peptides or proteins were protected from a lethal tumour challenge. A recent study investigated epicutaneous immunisation with the AntpSIIN OVA CD8 (AntpSIIN) fusion peptide, where topical application of AntpSIIN induced potent CTL responses in mice and with the adjuvant CpG conferred tumour protection against E.G7-OVA tumour cells [22]. Yet to date no study has directly compared the various CPP and their relative capacities to deliver tumour antigens and subsequent immunogenicity.

We have compared the efficiency of TAT and penetratin linked to either the H-2K^b^ CD8 8-mer epitope SIINFEKL from the model antigen ovalbumin (OVA) (TATSIIN, AntpSIIN), or to the H-2K^b^ CD8 9-mer epitope SAPDTRPAP from the human tumour associated antigen mucin 1 (MUC1) (TATMUC1K^b^, AntpMUC1K^b^). These studies showed that the tandem fusion peptide of Antp with SIINFEKL was immunogenic in mice, whereas TAT fused to SIINFEKL was not. In contrast, the immunogenicity of the MUC1 cytotoxic T cell epitope fused in tandem to either TAT or Antp CPP was identical.

## 2. Results

### 2.1. Stimulation of B3Z T Cells in Vitro by AntpSIIN and TATSIIN Pulsed DC

To establish the toxicity of Antp and TAT peptides on cells, AntpSIIN, TATSIIN, AntpMUC1K^b^ and TATMUC1K^b^ at varying concentrations were added to DC2.4 cells and cell death was measured quantitatively by lactate dehydrogenase (LDH) levels, a stable cytosolic enzyme that is released upon cell lysis. Cells exposed to Triton-X-100 were used as a positive control. None of these peptide antigens induced detectable levels of cell death when used at up to 200 µg/mL (not shown).

**Figure 1:**
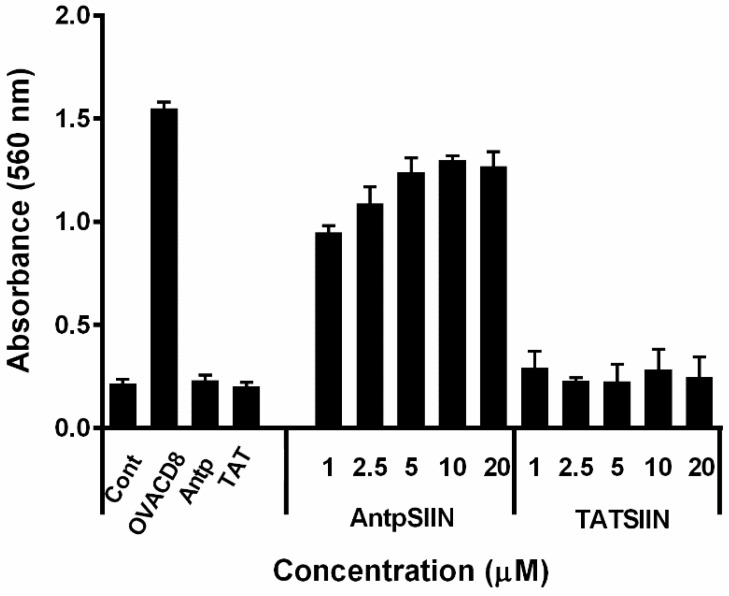
*In vitro* stimulation of T cells by AntpSIIN or TATSIIN pulsed bone marrow derived dendritic cells (BMDC). DC were incubated with AntpSIIN, TATSIIN, OVACD8, Antp, TAT peptide or media for 8 h and added to B3Z T cells for 18 h. LacZ activity in B3Z T cells was assayed by total culture lysates with LacZ substrate CPRG. The absorbance (560 nm) of chlorophenol red released by β-galactosidase was read after 4 h incubation at 37 °C. Values are the mean ± SEM for 4 replicates.

To compare the processing and presentation of AntpSIIN and TATSIIN, BMDC (bone marrow derived dendritic cells) were pulsed with varying peptide concentrations then incubated with B3Z T cells for 18 h. The recognition of the SIINFEKL epitope on the MHC class I molecule by its specific TCR was assessed via a colorimetric assay. Untreated DC and DC with Antp or TAT were used as negative controls. DC pulsed with AntpSIIN strongly presented SIINFEKL to B3Z T cells (Figure 1). Surprisingly and in contrast, DC pulsed with TATSIIN at 1 to 20 µM did not measurably activate T cells. DC pulsed with SIINFEKL peptide alone, which is surface loaded, was used as a positive control.

### 2.2. AntpSIIN but not TATSIIN Induce Potent in Vivo Proliferation and Killing

**Figure 2:**
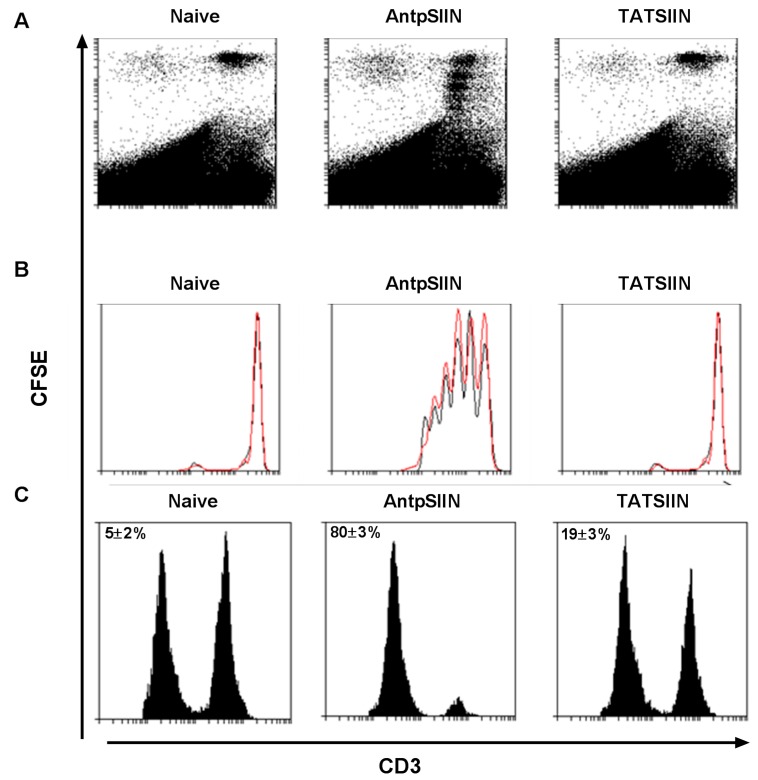
Measurement of *in vivo* CD3^+^ T cell proliferation and CTL lysis. C57BL/6 mice were immunised i.d. with PBS, AntpSIIN or TATSIIN and purified CFSE labelled OT-I strain T cells were injected i.v. Splenocytes were subsequently assessed via flow cytometry. CD3^+^ OT-I T cells that have undergone 0–5 cell divisions are shown as representative dot plots (**A**) and as CFSE profile histograms (**B**); with viable CD3^+^ OT-I T cells (black line) and CFSE curve fitting generated by the Weasel curve fitting software (red line); (**C**) The percentage of SIINFEKL specific lysis was determined in immunised mice by the *in vivo* killing assay 8 days later, shown by representative histograms and mean percent killing ± SEM (*n* = 6).

To assess the ability of AntpSIIN and TATSIIN to induce proliferation of T cells *in vivo*, mice were immunised with 25 µg AntpSIIN or TATSIIN and 20 h later were injected i.v. with purified OT-1 splenocyte T cells labelled with CFSE. 60 h after i.v injection, CD3^+^ splenocyte T cells were analysed by flow cytometry for antigen specific proliferation. Mice immunised with AntpSIIN demonstrated substantial T cell proliferation *in vivo*, seen as a sequential diminution of CFSE fluorescence in the daughter cells. However mice immunised with TATSIIN showed no proliferation greater than the control group (Figure 2A,B). To further investigate the *in vivo* T cell responses, the capacity of mice to generate SIINFEKL specific killing *in vivo* was assessed (Figure 2C). No SIINFEKL specific CTL response was detected in the spleens of control mice. Mice immunised with TATSIIN had evidence of weak antigen-specific killing (19% ± 3%), and much greater target cell killing (80% ± 3%) was detected in mice immunised with AntpSIIN (Figure 2C).

### 2.3. AntpSIIN but not TATSIIN Induce Strong OVA-Specific IFNγ T Cell Responses in Vivo

**Figure 3:**
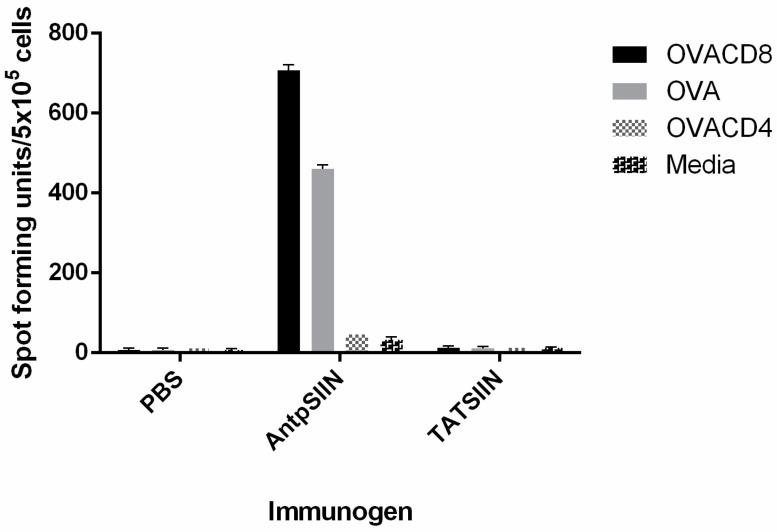
*In vivo* IFN-γ response to AntpSIIN and TATSIIN immunisation. C57BL/6 mice were injected i.d. on days 0, 10 and 17 with PBS, 25 µg AntpSIIN or 25 µg TATSIIN. The number of IFN-γ secreting cells in response to stimulation by OVACD8 (SIINFEKL), OVACD4 (OVA323-339) or media was analysed by ELISpot assay. Results are shown as mean spot-forming units (SFU)/5 × 10^5^ cells ± SEM in triplicate wells.

The ability of AntpSIIN and TATSIIN to induce CD8^+^ T cell responses *in vivo* was determined using an IFN-γ ELISpot assay. C57BL/6 mice were immunised with 25 µg AntpSIIN or TATSIIN on days 0, 10 and 17 and IFN-γ was measured 14 days later. Mice immunised with AntpSIIN generated potent IFN-γ secreting cells which recognised SIINFEKL as well as OVA (Figure 3). The CD4 epitope of OVA (OVA323-339) was used as a negative control. Mice immunised with TATSIIN or PBS did not generate measurable IFN-γ secreting cells to either SIINFEKL or OVA (Figure 3).

**Figure 4:**
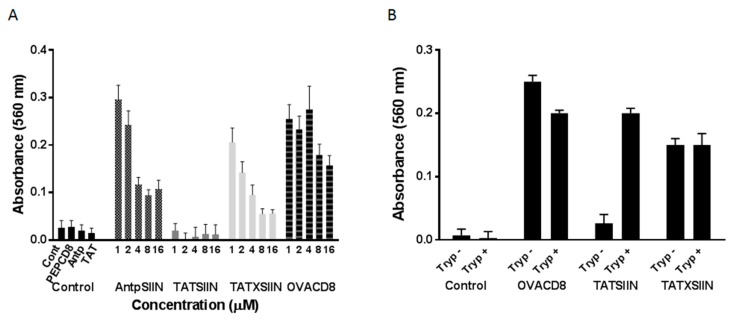
(**A**) *In vitro* stimulation of SIINFEKL-specific T cells by AntpSIIN, TATSIIN and TATXSIIN pulsed DC2.4. Cells were incubated with the peptide antigens for 8 h and added to B3Z T cells for 18 h. Controls included cells alone, Antp, OVACD8 (SIINFEKL) and PEPCD8, a non-internalising peptide; (**B**) *In vitro* stimulation of SIINFEKL-specific cells by DC2.4 cells pulsed with tryptic digests (Tryp) of TATSIIN, TATXSIIN and SIINFEKL. Cells were incubated with the tryptic digests as in A. In both (**A**) and (**B**), LacZ activity in B3Z T cells was assayed by total culture lysates with LacZ substrate CPRG. The absorbance (560 nm) of chlorophenol red released by β-galactosidase was read after 4 h incubation at 37 °C. Values show the mean ± SEM for 4 replicates.

To ascertain if the lack of SIINFEKL-specific responses *in vitro* and *in vivo* was due to an inability of TATSIIN to be processed appropriately a similar peptide (TATXSIIN), incorporating a linker of 4 amino acids preceding SIINFEKL in the native OVA sequence, was used in the B3Z assay. When the peptide was incubated with DC2.4 cells *in vitro* it was processed and stimulated the SIINFEKL-specific B3Z T cells. A control peptide (PEPCD8) of equal length to TATSIIN, incorporating only the amino acids preceding SIINFEKL in the native OVA sequence, was not efficiently presented to B3Z cells by DC2.4 cells (Figure 4). To demonstrate that the SIINFEKL epitope of TATSIIN can be presented by DC2.4 if correctly processed, the enzyme trypsin that cleaves at arginine and lysines present in the TAT sequence was used. DC2.4 cells incubated with a tryptic digest of TATSIIN efficiently activated B3Z cells by surface loading the class I molecules with enzymatically released SIINFEKL (Figure 4B). Similarly tryptic digests of TATXSIIN and SIINFEKL also activated B3Z cells indicating the resistance of SIINFEKL epitope to trypsin.

### 2.4. Rapid Internalisation of Antp and TAT Peptides Incorporating MUC1-H-2K^b^ Epitope

Several mouse and human CTL epitopes of MUC1 that can be presented by particular human and mouse MHC molecules have been identified [23,24,25,26]. The peptide SAPDTRPAP, presented by mouse H-2K^b^, was used to study the comparative immunogenicity of TAT and Antp-based cytotoxic T cell epitopes. An additional advantage of this epitope is that the DTR sequence within the cytotoxic T cell epitope contains a linear B-cell epitope and the monoclonal antibody BC2 recognises the DTR sequence enabling detection of internalised AntMUC1K^b^ or TATMUC1K^b^ peptide without modification.

**Figure 5:**
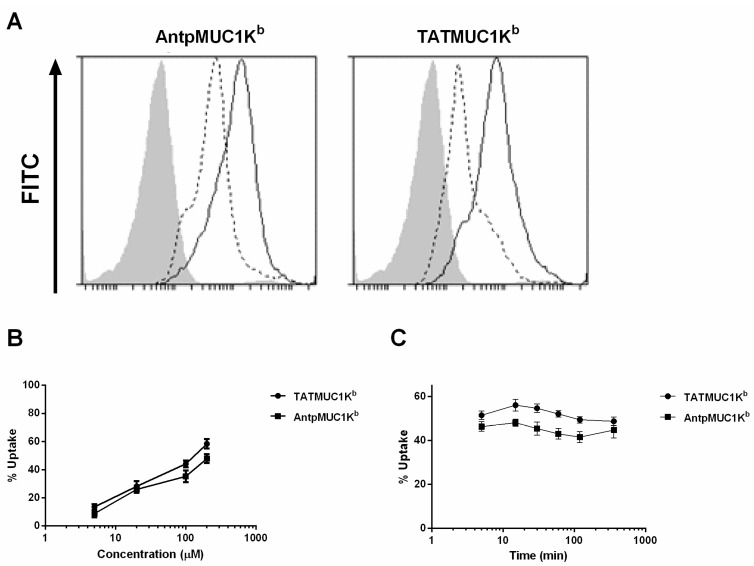
Uptake of AntpMUC1K^b^ and TATMUC1K^b^ peptides by DC *in vitro*. (**A**) AntpMUC1K^b^ and TATMUC1K^b^ staining by BC2 antibody after pulsing at 100 µM for 60 min with uptake assessed as the difference between surface (dotted line) and intracellular (bold line) staining by flow cytometry. Isotype controls are shown as filled grey areas. DC were pulsed with AntpMUC1K^b^ or TATMUC1K^b^ peptides at either varying concentrations (5 to 200 µM) for 60 min (**B**) or a constant dose of 100 µM for set times between 5 and 360 min (**C**). Uptake was determined by flow cytometry as the percent surface staining subtracted from the percent intracellular staining (mean ± SEM, for 3 replicates).

The mechanism of cellular uptake of CPP has been an area of great conjecture over recent years [27,28,29]. Cell fixation leads to the artifactual uptake of these peptides, thus, to assess the mechanism of uptake of AntpMUC1K^b^ and TATMUC1K^b^ by DC, surface and intracellular staining of the antibody BC2 was measured by flow cytometry. BMDC pulsed with AntpMUC1K^b^ or TATMUC1K^b^ showed surface binding of 11% and 51%, respectively, and substantial peptide internalisation of 48% and 90%, respectively (Figure 5A).

To analyse the dose response and kinetics of uptake of AntpMUC1K^b^ and TATMUC1K^b^, BMDC prepared *in vitro* were pulsed with 5, 20, 100 or 200 µM AntpMUC1K^b^ or TATMUC1K^b^ peptides for 60 min. The percent positive cells at the low concentration of 5 µM AntpMUC1K^b^ or TATMUC1K^b^ was 9% and 13% respectively, reaching 48% and 58% respectively at the high concentration of 200 µM (Figure 5B). To address the kinetics of uptake, BMDC were incubated for set times with AntpMUC1K^b^ or TATMUC1K^b^ peptides at a fixed concentration (100 µM). Both AntpMUC1K^b^ and TATMUC1K^b^ displayed similar uptake kinetics with rapid internalisation seen within 5 min which remained constant for at least 360 min (Figure 5C).

**Figure 6:**
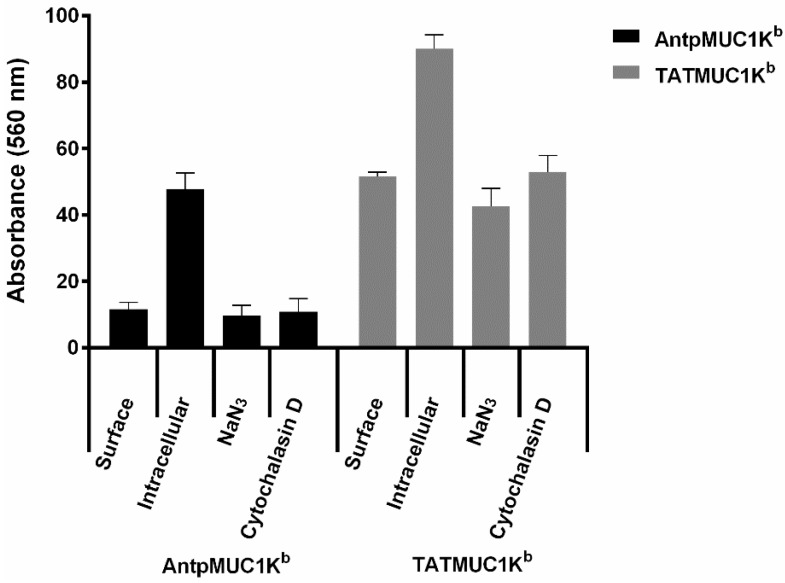
Uptake of AntpMUC1K^b^ and TATMUC1K^b^ by BMDC is via endocytosis. DC cultures were pre-treated for 45 min with cytochalasin D (10 µg/mL) or NaN_3_/2-deoxyglucose (10 mM) before adding 100 µg/mL of AntpMUC1K^b^ or TATMUC1K^b^ for 60 min. Uptake was determined via flow cytometry and expressed as the percent surface staining subtracted from the percent intracellular staining (mean ± SEM, for 3 replicates).

To investigate if internalisation was via an energy dependant pathway, uptake was measured in the presence of NaN_3_/2-deoxyglucose and cytochalasin D. NaN_3_/2-deoxyglucose (10 mM) depletes ATP-dependent mechanisms in the cell and blocks cell membrane activity while Cytochalasin D (10 µg/mL) affects the contraction of actin containing microfilaments and blocks phagocytosis. Both Cytochalasin D and NaN_3_/2-deoxyglucose blocked the uptake of both AntpMUC1K^b^ and TATMUC1K^b^ by DC *in vitro* (Figure 6), indicating that it is by endocytosis.

### 2.5. AntpMUC1K^b^ and TATMUC1K^b^ Induce Potent in vivo Cytotoxic T Cell Killing

**Figure 7:**
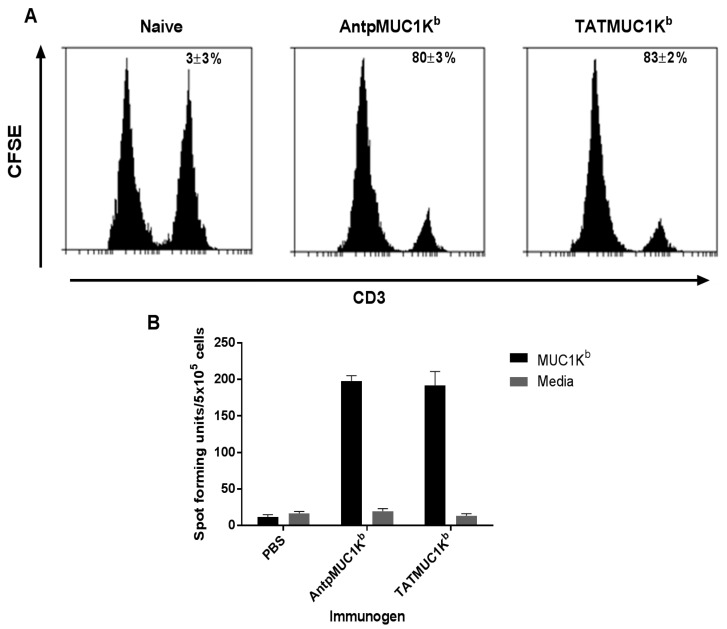
Cellular immune responses in TATMUC1K^b^ and AntpMUC1K^b^ immunised mice, measured as *in vivo* CTL killing and ELISpot assays. (**A**) C57BL/6 mice were immunised i.d. with PBS, 25 µg AntpMUC1K^b^ or 25 µg TATMUC1K^b^ and the percent MUC1 SAPDTRPAP specific lysis was determined eight days after immunisation. Representative histograms from 2 mice are shown; (**B**) IFN-γ responses to AntpMUC1K^b^ and TATMUC1K^b^ peptides in C57BL/6 mice, injected as above, with the number of IFN-γ secreting cells analysed by ELISpot assay. Results are shown as mean spot-forming units (SFU)/5 × 10^5^ cells ± SEM with 3 replicates. Results are representative of two experiments.

The capacity of C57BL/6 mice to generate SAPDTRPAP antigen specific killing *in vivo* was assessed. Splenocytes from naïve mice were prepared, pulsed with SAPDTRPAP peptide and labelled with CFSE at high or low concentrations, respectively. Cells were subsequently adoptively transferred into mice pre immunised with either 25 µg AntpMUC1K^b^ or TATMUC1K^b^ and the percent killing was determined. No SAPDTRPAP CTL responses were detected in the spleen of control mice (Figure 7A). In contrast, strong *in vivo* killing was detected when mice were immunised with either AntpMUC1K^b^ (80% ± 3%) or TATMUC1K^b^ (83% ± 2%), with no difference in efficacy observed (*p* > 0.05).

### 2.6. AntpMUC1K^b^ and TATMUC1K^b^ Immunisation Induces Strong SAPDTRPAP T Cell Specific IFN-γ

To further evaluate the *in vivo* immune responses, the capacity of AntpMUC1K^b^ and TATMUC1K^b^ antigens to induce CD8^+^ T cell responses *in vivo* was determined using IFN-γ ELISpot analysis. C57BL/6 mice were immunised i.d. with 25 µg AntpMUC1 K^b^ or TATMUC1K^b^ on days 0, 10 and 17 and IFN-γ was measured 14 days later. Mice immunised with either AntpMUC1K^b^ or TATMUC1K^b^ generated equally strong IFN-γ secreting cell responses to the SAPDTRPAP (MUC1 K^b^) epitope (Figure 7B).

The capacity of AntpMUC1K^b^ and TATMUC1K^b^ antigens to generate antibody responses to MUC1 was assessed via ELISA. At the time of the ELISpot assay, sera were collected to determine the total IgG isotype responses. Mice immunised with AntpMUC1K^b^ or TATMUC1K^b^ did not generate antibody titres significantly greater than that of control mice (not shown).

### 2.7. Mice Immunised with AntpMUC1K^b^ and TATMUC1K^b^ Inhibited MUC1^+^ Tumour Growth in C57BL/6 Mice

**Figure 8:**
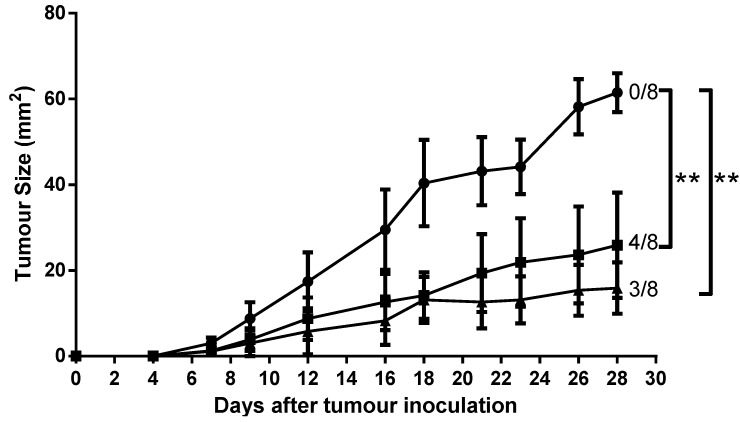
Tumour growth is delayed by immunisation. C57BL/6 mice were immunised on days 0, 10 and 17 with PBS, 25 µg AntpMUC1K^b^ or 25 µg TATMUC1K^b^ then inoculated subcutaneously 7 days after final immunisation with 2 × 10^5^ B16-MUC1 melanoma cells into the abdomen. Tumour growth was recorded. Data showing the product of individual perpendicular measurements (mm^2^) and days post tumour inoculation. Number of tumour-free mice at day 28 is also shown (*n* = 8/group, ** *p* < 0.05).

To assess whether AntpMUC1K^b^ or TATMUC1K^b^ immunisation confers tumour protection *in vivo,* C57BL/6 mice (*n* = 8) were injected i.d. with PBS, 25 µg AntpMUC1K^b^ or 25 µg TATMUC1K^b^ on days 0, 10 and 17. Seven days later, mice were challenged s.c. with 2 × 10^5^ B16-MUC1 tumour cells. Immunisation of mice with either AntpMUC1K^b^ or TATMUC1K^b^ delayed tumour growth (Figure 8). On day 28 the average tumour sizes in mice immunised with AntpMUC1K^b^ or TATMUC1K^b^ were 25.9 and 15.9 mm^2^, respectively, significantly less than 61.5 mm^2^ in control mice immunised with PBS (*p* < 0.05). In addition 4 of 8 AntpMUC1immunised mice and 3 of 8 TATMUC1 immunised mice were tumour free at day 28.

## 3. Discussion

Peptide and protein based vaccines based solely on CTL epitopes have limited efficacy, as APC are not efficient in the uptake and processing of exogenous antigens via the MHC class I pathway [1,2,3]. Thus, novel methods to enhance antigen delivery into APC are required. CPPs are of special interest in vaccine design due to their capacity to facilitate the delivery of therapeutic substances into cellular compartments [28,29,30]. Thus CPP provide an effective means to facilitate intracellular delivery of antigens to APC and induction of a CTL response [18]. The ability of tumour antigens or their T cell epitopes when linked to CPP to enhance immunogenicity has been established. Kim *et al.* were the first to demonstrate that immunisation with DCs pulsed with TAT peptide conjugated OVA induced antigen specific CTLs *in vivo*, whereas DC pulsed with full length OVA protein failed to promote antigen specific cytotoxicity [31]. Several studies characterised the potent immunogenicity of conjugates of TAT or Antp CPP with proteins or peptides or recombinant proteins with a CPP linked in tandem. Intraperitoneal immunisation with a synthetic peptide incorporating Antp and OVA CD8 epitope was shown to generate potent CTL responses and protective immunity *in vivo* against the growth of the OVA expressing tumour cell line E.G7-OVA [14]. Additionally i.d. immunisation was shown to be as effective, with mice generating potent CD8^+^ specific IFN-γ responses to immunisation with AntpSIIN alone or AntpSIIN pulsed DC, and protection against the growth of OVA expressing tumours. Moreover, similar IFN-γ responses were observed following a single immunization [15]. Recently we demonstrated the immunogenicity and tumour protection of mice immunised with covalent conjugates of Antp with OVA and synthetic peptides of Antp linked in tandem to OVA CD8, CD4 and CD8/CD4 epitopes [16].

There is a large amount of data suggesting that various CPP function differently and are influenced by the cargo being delivered or the cell type. This study compared the immunogenicity of synthetic peptides incorporating either the H-2K^b^ CTL epitope from the model antigen OVA SIINFEKL or the MUC1K^b^ SAPDTRPAP epitope linked in tandem to the TAT peptide or to Antp. When linked to the TAT peptide, SIINFEKL (TATSIIN) failed to be processed and presented to T cells either *in vitro* or *in vivo* (Figure 1, Figure 2 and Figure 3). In contrast, the synthetic peptide of Antp linked to SIINFEKL (AntpSIIN) generated a strong *in vivo* immune response (Figure 3). Synthetic peptides of MUC1K^b^ SAPDTRPAP epitope with either TAT or Antp were both rapidly and efficiently internalised by DC in an ATP dependant pathway via macropinocytosis or phagocytosis (Figure 5 and Figure 6). Furthermore, mice immunised with either AntpMUC1K^b^ or TATMUC1K^b^ peptides generated strong MUC1-specific IFN-γ responses and inhibited the growth of B16-MUC1 tumours (Figure 7 and Figure 8).

It was interesting that the TATSIIN peptide, sequence RKKRRQRRRSIINFEKL without a linker between TAT and SIINFEKL, was not processed and presented by DC to T cells *in vitro* or *in vivo,* whilst TATMUC1K^b^, AntpSIIN and AntpMUC1K^b^ were effective. Studies that have assessed TAT to facilitate the delivery of the SIINFEKL epitope have incorporated a sequence to facilitate epitope cleavage. Lu and colleagues utilized the 9-mer TAT (RKKRRQRRR) peptide and furin sensitive (RVKR) or furin insensitive (VRVV) linkers to multiple CTL epitopes [32]. Furin is a type I membrane protein localised predominantly in the Golgi complex, but also found at the plasma membrane and within endosomes. Furin plays a role in the endoproteolytic processing of proteins within the Golgi and furin cleavage is known to be required for endosome escape by several bacterial toxins, including Anthrax toxin and Pseudomonas exotoxin A. It was observed that TAT peptides with furin sensitive linkers sensitised target cells for CTL lysis whereas furin insensitive linkers failed to prime a response [32]. To demonstrate that presentation was inefficient or absent for TATSIIN, we synthesised a peptide incorporating a linker consisting of amino acids preceding the SIINFEKL epitope from the native OVA sequence. This novel peptide was efficiently processed by DC and presented to the B3Z OVA-specific T cell hybridoma (Figure 4A). Trypsin digestion of TATSIIN and TATXSIIN released the SIINFEKL epitope which was functional in stimulating B3Z cells(Figure 4B) indicating that correct processing of peptide would have resulted in proper presentation. In addition this experiment indicates that inability of TATSIIN to activate SIINFEKL-specific T cells is not due to instability of the peptide.

The uptake and intracellular processing pathways of Antp and TAT, when linked to a single CTL epitope, has been extensively characterised. Using primary bone marrow-derived mouse DCs, AntpSIIN was shown to be endocytosed in an ATP dependent manner with the involvement of negatively charged receptors. Further investigation revealed the majority of peptide was taken up via phagocytosis and/or macropinocytosis in a caveolae independent manner [15]. Examination of the intracellular pathways revealed processing via a proteasome independent pathway through endosomes and lysosomes in a TAP-independent process to the ER for presentation by MHC Class I molecules. No trimming by furin endopeptidase in the trans-Golgi or by aminopeptidases was required [15]. Likewise TAT linked to CTL epitopes by a triple alanine spacer was found to be processed via a TAP independent pathway, through the Golgi and ER [33]. Immunofluorescence studies had demonstrated the rapid uptake of AntpMUC1K^b^, AntpMUC1A2 and AntpMUC1FP peptide and proteins into the cytoplasm of peritoneal macrophages, whereas proteins and peptides without the internalising sequence were not efficiently taken up [11]. Flow cytometry studies reported here indicated that TAT and Antp promoted similar levels of uptake into DC by an ATP dependant process involving phagocytosis and/or micropinocytosis (Figure 5 and Figure 6).

Mice immunised with either AntpMUC1K^b^ or TATMUC1K^b^ peptides generated potent cellular responses, measured by *in vivo* CTL killing and IFN-γ ELISpot assays, however neither AntpMUC1K^b^ nor TATMUC1K^b^ immunisation induced an antibody response (Figure 7). Investigations of a recombinant fusion protein consisting of the 60-amino acid Antennapedia homeodomain fused to an influenza derived HLA-Cw3-restricted CTL epitope required either SDS as an adjuvant to prime a CTL response or incorporation into liposomes enhanced by the addition of CpG. Likewise, vaccination with a vaccine incorporating TAT linked to antigens from OVA required CpG to generate an anti-tumour response [33]. However here we report a delayed tumour response after immunisation with either AntpMUC1K^b^ or TATMUC1K^b^ without the need for an adjuvant (Figure 8). It was previously demonstrated that MUC1K^b^ immunisation alone, without the addition of CPP or other carriers, did not confer protection against a tumour challenge [34].

In conclusion, this study demonstrated that the TAT protein linked to SIINFEKL, without an intermediate linker sequence, failed to be processed and presented to T cells either *in vitro* or *in vivo.* In contrast, linking Antp to SIINFEKL (AntpSIIN) generated strong immune responses. Fusion of the MUC1K^b^ SAPDTRPAP epitope to either TAT or Antp demonstrated that both are rapidly and efficiently internalised by BMDC in an ATP dependant manner. Furthermore, mice immunised with either AntpMUC1K^b^ or TATMUC1K^b^ generated strong *in vivo* IFN-γ T cell responses and showed delayed B16-MUC1 tumour growth. Most importantly, these studies indicated that TAT and Antp both function equivalently for delivery of cytotoxic T cell epitopes to APC, provided a suitable linker is used.

## 4. Experimental Section

### 4.1. Peptides

Peptides (Table 1) were synthesized by Genescript Corporation (San Francisco, CA, USA). Molecular weights were confirmed by MS and had purities of >98% by HPLC.

**Table 1:** Synthetic peptides used in the study.

Peptide Name	Sequence	Description
Antp	*RQIKIWFQNRRMKWKK*	16 amino acid *Antennapedia* peptide
TAT	*RKKRRQRRR*	9-mer HIV TAT protein transduction domain
OVACD8	SIINFEKL	ovalbumin H-2K^b^ CTL epitope 8-mer peptide.
OVACD4	ISQAVHAAHAEINEAGR	ovalbumin IA^b^ CD4 epitope 16-mer peptide (OVA323-339)
MUC1K^b^	SAPDTRPAP	Mucin 1 H-2K^b^ epitope from the VNTR region.
AntpSIIN	*RQIKIWFQNRRMKWKK*SIINFEKL	C-terminal fusion peptide of Antp and OVACD8
TATSIIN	*RKKRRQRRR*SIINFEKL	C-terminal fusion peptide of Tat and OVACD8
AntpMUC1K^b^	*RQIKIWFQNRRMKWKK*SAPDTRPAP	C-terminal fusion peptide of Antp and MUC1K^b^
TATMUC1K^b^	*RKKRRQRRR*SAPDTRPAP	C-terminal fusion peptide of Tat and MUC1K^b^
PEPCD8	LLPDEVSGLEQLESIINFEKL	Non-internalising control peptide including 13 amino acids N-terminal to SIINFEKL in native OVA sequence
TATXSIIN	*RKKRRQRRR*EQLESIINFEKL	N-terminal fusion peptide of Tat and OVACD8 including 4 aa native sequence

### 4.2. Mice

C57BL/6 and OT-I mice, aged 6–10 weeks, were purchased from the Biological Research facilities of the Walter and Eliza Hall Institute (Parkville, Australia). All mice were housed in the facilities at Burnet Institute (Heidelberg Campus) or RMIT University.

### 4.3. Cytotoxicity of Conjugates

DC2.4 cells (10^4^) were seeded in a volume of 100 µL complete RPMI media (10% (*v*/*v*) heat inactivated fetal calf serum, 4 mM L-glutamine, 100 U/mL penicillin, 100 µg/mL streptomycin sulphate and 100 µM β-mercaptoethanol) in a flat bottom 96-well microtitre plate and allowed to adhere overnight at 37 °C. Next day media was removed and replaced with 200 µL complete RPMI with peptides at specified concentrations. Cells were incubated for 24 h at 37 °C and subsequently 100 µL media was removed to determine cytotoxicity with the CytoTox 96 Non-Radioactive Cytotoxicity Assay according to manufacturer’s instructions (Promega, Madison, WI, USA). 50 µL/well of reconstituted substrate mix was added to the enzymatic assay plate containing samples and incubated for 30 min at RT. 50 µL/well of stop solution was added and absorbance read at 490 nm.

Cytotoxicity was calculated as:

(1) $$\%Cytotoxicity=\frac{OD_{490nm}\left(experimental\;LDH\;release\right)}{OD_{490nm}\left(maximum\;LDH\;release\right)}\times 100$$

### 4.4. Generation of Bone-Marrow Derived Dendritic Cells (BMDC)

Bone marrow cells from C57BL/6 female mice were collected by flushing the tibias of hind legs and treated with ACK lysis buffer (0.15 M NH_4_Cl, 1 mM KHCO_3_, 0.1 mM Na_2_EDTA) to lyse erythrocytes. Cells were washed and cultured at 5 × 10^5^ cells/mL in 24 well plates with complete RPMI-1640 medium with 10 ng/mL of recombinant mouse granulocyte macrophage colony-stimulating factor (GM-CSF) (BD Pharmingen, San Diego, CA, USA). At day 6, cells were observed to be >80% CD11c^+^ by flow cytometry.

### 4.5. Internalisation into BMDC

Day 6 cultured C57BL/6 BMDC were pulsed with 5, 20, 100 or 200 µM peptides for 1 h at 37 °C in serum free media. Due to cell fixation causing artifactual uptake of CPP peptides, all uptake experiments were performed by measuring surface and intracellular expression by flow cytometry, with results expressed as percent intracellular stain–percent surface stain. For surface staining, DC were washed with 0.5% *w*/*v* BSA/PBS and incubated with the anti-MUC1 monoclonal antibody BC2 (diluted in BSA/PBS) for 30 min at 4 °C. Cells were washed and FITC-anti-mouse (Fab’)_2_ (Chemicon, Melbourne, Australia) was added in BSA/PBS for a further 30 min at 4 °C. For intracellular staining, cells were fixed with 2% paraformaldehyde for 10 min at room temperature, washed and permeabilised with 0.25% *v*/*v* saponin/PBS for 10 min. Cells were then stained as above in saponin/PBS. Kinetics of uptake was also assessed by adding peptides at a fixed concentration for specified times (5 to 360 min) at 37 °C. DC were resuspended in PBS and analysed by flow cytometry (BDCanto, BD Biosciences, San Jose, CA, USA). Results are expressed as percent intracellular stain–percent surface stain. Isotype controls were used to measure negative control (background) staining.

### 4.6. Internalisation Inhibition Studies

BMDC were pre incubated with and without biochemical inhibitors; 10 mM sodium azide (NaN_3_) (Sigma, Suffolk, UK) and 2-deoxyglucose (2DG) (Sigma) or 10 µg/mL cytochalasin D (Sigma), which blocks phagocytosis, for 45 min at 37 °C. Cells were then pulsed with AntpMUC1K^b^ or TATMUC1K^b^ for 60 min at 20 µg/mL at 37 °C. Uptake of antigen was measured as described in Section 4.5.

### 4.7. Stimulation of lacZ-Inducible Ovalbumin-Specific T-cell Hybrid Cells

The B3Z mouse T-cell hybridoma line contains a gene construct of *Escherichia coli* lacZ reporter gene linked to the nuclear factor of activated T cells. Recognition of the SIINFEKL peptide in the context of class I by the T-cell receptor (TCR) results in activation of the enzyme and conversion of a chromogenic substrate that can be measured by absorbance spectrophotometry. DC (2 × 10^5^ cells) were pulsed with peptides at various concentrations in 96-well microtitre plates (Falcon, BD Biosciences, North Ryde, Australia) for 24 h at 37 °C. Cells were then washed and 10^5^ B3Z cells were added for 18 h at 37 °C. Next day, cells were washed with sterile PBS and incubated with chlorophenol red-β-galactoside (Calbiochem, San Diego, CA, USA) (100 µM 2ME, 9 mM MgCl_2_, 0.125% NP40, 0.15 mM chlorophenol red-β-galactoside). After 4 h incubation at 37 °C the absorbance was read at 560 nm to detect chlorophenol red released by β-galactosidase.

### 4.8. In vivo Proliferation

Splenocytes from OT-I mice were isolated and purified as described in section 4.4. Purified OT-I T cells were resuspended in 0.1% *w*/*v* BSA/PBS and labelled with 5 μM carboxyfluorescein succinimidyl ester (CFSE) (Molecular Probes) for 10 min at 37 °C. Labelling was stopped with a 5× volume of ice cold complete RPMI and cells were washed extensively in PBS. CFSE labeling was confirmed by flow cytometry. 10^7^ CFSE labelled OT-I T cells were then injected intravenously (i.v.) in 200 µL PBS into C57BL/6 mice immunised i.d. at the base of the tail 20 h prior. Splenocytes were collected 60 h following CFSE injection and stained with APC-conjugated anti-CD3 (BD Pharmingen) for 30 min at 4 °C in 2% FCS/PBS. Cells were washed and CFSE dilution was determined by flow cytometry and analysed by Weasel curve fitting software (version 2.4, Walter and Eliza Hall Institute).

### 4.9. In vivo Cytotoxicity Assay

Splenocytes from naïve C57BL/6 mice were isolated and resuspended to 10^7^ cells/mL in serum free RPMI and divided into 2 populations, pulsed or unpulsed. Pulsed splenocytes were incubated with 1 µg/mL SIINFEKL or SAPDTRPAP peptide for 1 h at 37 °C. Cells were washed and resuspended in 0.1% *w*/*v* BSA/PBS and pulsed splenocytes were labelled with a high concentration of CFSE (5 µM) whereas unpulsed splenocytes were labelled with a concentration of CFSE (0.5 µM) for 10 min at 37 °C. Labelling was stopped with 5× volumes of ice cold complete RPMI and cells were washed extensively in PBS.

Peptide-pulsed 5 × 10^6^ CFSE^high^ cells and 5 × 10^6^ unpulsed CFSE^low^ cells were mixed and a total of 10^7^ CFSE labelled cells in 200 µL PBS was injected i.v. into mice immunised 8 days prior or into naïve mice. After 20 h, splenocytes were isolated and analysed by flow cytometry.

Specific lysis was calculated as:

Specific lysis = 1 − {[ratio CFSE^low^/CFSE^high^ of naïve mice]/[ratio CFSE^low^/CFSE^high^ of immunised mice]} × 100 (2)

### 4.10. Enzyme-Linked Immunosorbent Spot-Forming Cell Assay (ELISpot)

To determine the effector immune response, splenocytes from C57BL/6 mice immunised i.d. on days 0, 10 and 17 were isolated 14 days after final immunisation and assessed by ELISpot for IFN-γ secretion. MultiScreen filter plates (Millipore, Billerica, MA, USA) were coated with 70 µL 5 µg/mL anti-mouse IFN-γ antibody (AN18) (Mabtech, Stockholm, Sweden) overnight at 4 °C. Plates were washed six times with sterile PBS and blocked with 200 µL complete RPMI media for 2 h at 37 °C. Spleen cells (5 × 10^5^/well) in 100 µL of complete medium were incubated with 20 µg/mL recall antigens for 18 h in IFN-γ ELISpot. Recall antigens were SIINFEKL (OVACD8, OVA257-264), ISQAVHAAHAEINEAGRKG (OVACD4, OVA323-339), OVA and SAPDTRPAP (MUC1K^b^). Concanavalin A (ConA) (1 µg/mL) or cells alone were used as positive and negative controls respectively. Triplicate wells were set up for each condition. Cells were discarded after washing (PBS) and 1 µg/mL biotinylated anti-mouse IFN-γ antibody (Mabtech) was added for 2 h at RT. The plates were washed with PBS and 1 µg/mL streptavidin-alkaline phosphatase (Mabtech) was added at room temperature for 2 h. Spots of activity were detected using a colorimetric AP-conjugate substrate kit (Bio-Rad Laboratories, Foster City, CA, USA). Cytokine spots were counted with an AID ELISpot Reader system (Autoimmun Diagnostika GmbH, Strassberg, Germany). Data were presented as mean spot-forming units (SFU) per 5 × 10^5^ cells ± standard error of the mean (SEM).

### 4.11. Antibody ELISA

Sera were collected from C57BL/6 mice 14 days after final immunisation by orbital bleed. Red blood cells were pelleted by centrifugation at 13,000 rpm for 10 min and serum was aspirated and stored at −20 °C until use.

The MUC1 peptide corresponding to a single VNTR repeat of MUC1, Cp13-32 (C-PAHGVTSAPDTRPAPGSTAP), was coated onto PVC microtiter plates at 10 µg/mL in 0.2M NaHCO_3_ buffer, pH 9.6, and overnight at 4 °C. After washing (0.05% Tween 20/PBS), non-specific binding was blocked with 2% BSA/PBS for 1 h at RT. Serial dilutions of sera were added (in 2% BSA/PBS) and incubated for a further 2 h at RT. Plates were washed and bound antibody was detected using horseradish peroxidase-conjugated sheep anti-mouse IgG (Amersham, UK). Plates were washed as described above and HRP-streptavidin was added for 1 h at RT. Responses were detected with TMB substrate solution and stopped with 1 M HCl. Absorbance was read at 450 nm.

### 4.12. Prophylactic Tumour Protection

Groups of 8 C57BL/6 mice were injected intradermally (i.d.) at the base of tail with 25 µg of various peptides or PBS on days 0, 10 and 17. Seven days following the last immunisation, mice were shaved on the abdominal area and challenged subcutaneously (s.c.) with 2 × 10^5^ MUC1-B16 melanoma cells suspended in 100 µL PBS. Expression of MUC1 on MUC1-B16 cells was confirmed by flow cytometry prior to challenge. The growth of tumours was monitored by measuring the two perpendicular diameters using callipers and the results were expressed as their product.

### 4.13. Statistical Analysis

Assays were set up in triplicate. Mean values were compared using the two-tailed unpaired t-test and ANOVA. Two p-value thresholds were used for protection and immunogenicity assays: *p* < 0.001 to indicate a highly significant difference, and *p* < 0.05 to indicate a significant difference.
